# Supplementary material for: Differentiating interfacial water structures via alkali metal cation promotor for H2O2 electrosynthesis in acid
Source: Nat Commun. 2026 Apr 8;17:4973. doi: 10.1038/s41467-026-71584-9 (PMC13237355; doi:10.1038/s41467-026-71584-9)
Supplement: Supplementary file 1 — Supplementary Information [file 41467_2026_71584_MOESM1_ESM.pdf]

Supplementary Information

Differentiating interfacial water structures via alkali metal cation promotor for H_2O_2 electrosynthesis in acid

Yifei Wang[1]*, Peiyang Duan[1], Yingqi Liao[2], Hao Wang[1], Beibei Li[1], Hangyuan Zhang[3], Hao Yang[2]*, Tao Cheng[2]* & Jingyu Sun[3]*

[1]National Engineering Laboratory for Advanced Municipal Wastewater Treatment and Reuse Technology, Key Laboratory of Beijing for Water Quality Science and Water Environment Recovery Engineering, Beijing University of Technology, Beijing, China.

[2]Institute of Functional Nano & Soft Materials, Jiangsu Provincial Key Laboratory for Carbon-Based Functional Materials & Devices, Joint International Research Laboratory of Carbon-Based Functional Materials and Devices, Soochow University, Suzhou, China.

[3]College of Energy, Soochow Institute for Energy and Materials Innovations, Key Laboratory of Advanced Carbon Materials and Wearable Energy Technologies of Jiangsu Province, Soochow University, Suzhou, China.

*Corresponding authors: wangyifei@bjut.edu.cn; haoyang@suda.edu.cn; tcheng@suda.edu.cn; sunjy86@suda.edu.cn.

Supplementary Figure

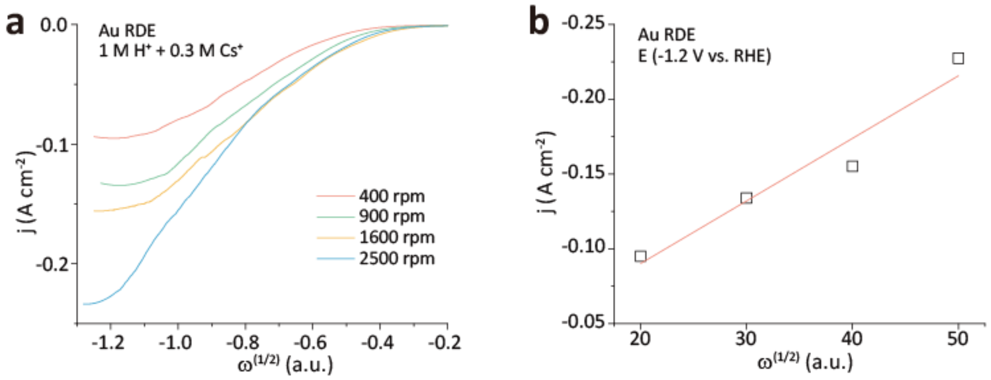

Supplementary Fig. 1 | (a) HER Polarization curves of normally-sized Au rotating disk electrode (RDE, D = 5 mm) in 1 M H^+ + 0.3 M Cs^+ at 0.02 V s^{-1}. Rotating speed was set from 400 to 2500 rpm. (b) Linear relationship between the diffusion-limited current and square root of rotating speed for HER on the RDEs of Au, revealing the HER current was entirely mass-transport-limited by proton diffusion. Source data for Supplementary Fig. 1 are provided as a Source Data file.

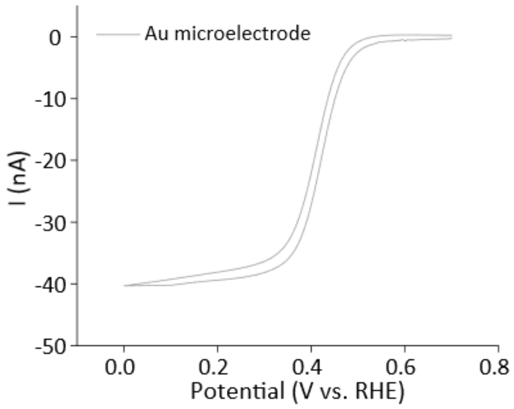

Supplementary Fig. 2 | CV curves for Au microelectrode in Ar-saturated 10 mM $[Ru(NH_3)_6]^{3+}$ + 0.1 M K_2SO_4 solution at a scan rate of 0.02 V s^{-1}. The measured radius of the Au microelectrode was 17.6 μm. Source data for Supplementary Fig. 2 are provided as a Source Data file.

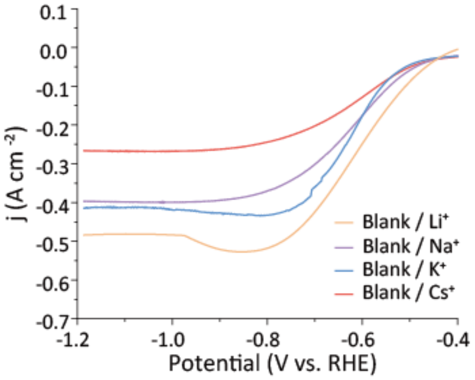

Supplementary Fig. 3 | HER polarization curves for Au microelectrodes in Ar-saturated 0.5 M H_2SO_4 + 0.15 M A_2SO_4 at 0.02 V s^{-1} (A = Li, Na, K, or Cs). Source data for Supplementary Fig. 3 are provided as a Source Data file.

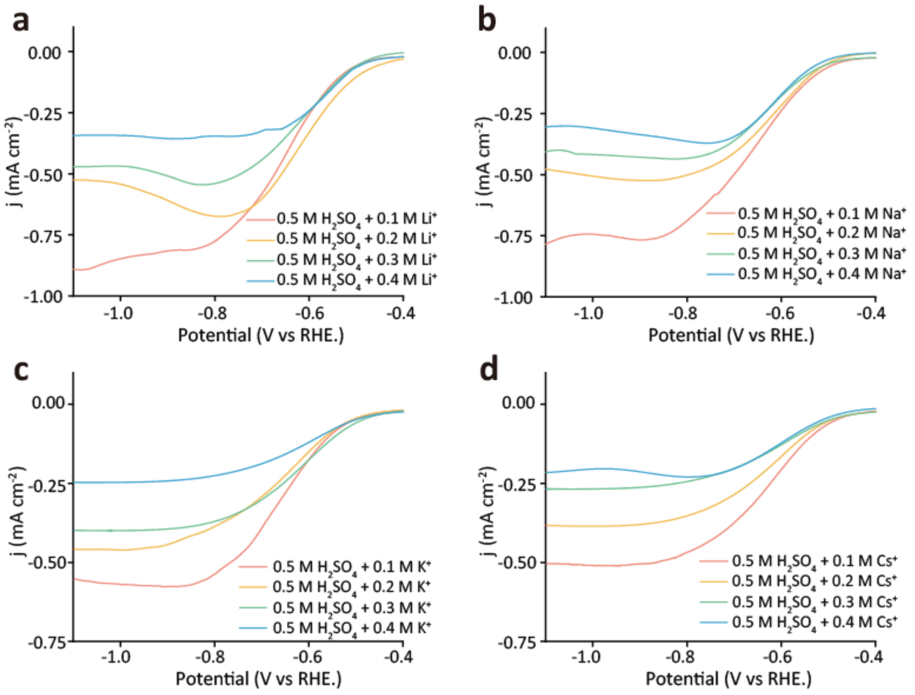

Supplementary Fig. 4 | HER polarization curves of Au microelectrodes in 0.5 M H_2SO_4 + *x* M Li^+/Na^+/K^+/Cs^+ (*x* ranging from 0.1 to 0.4) at 0.02 V s^{-1}. Source data for Supplementary Fig. 4 are provided as a Source Data file.

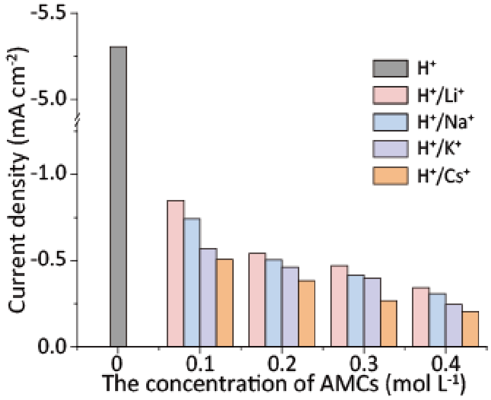

Supplementary Fig. 5 | Diffusion-limited current density for 1 M H^+ and 1 M H^+ + *x* M A^+ (*x* ranging from 0.1 to 0.4; A = Li, Na, K, or Cs). Source data for Supplementary Fig. 5 are provided as a Source Data file.

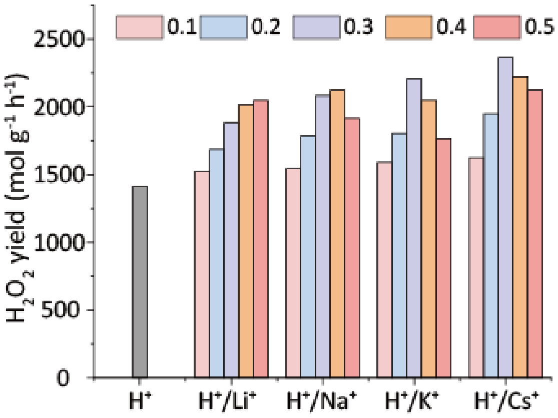

Supplementary Fig. 6 | H_2O_2 yield based on Co-CNT at different AMC concentrations in a flow-cell reactor. Source data for Supplementary Fig. 6 are provided as a Source Data file.

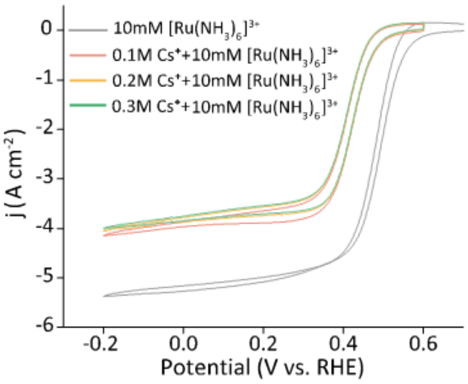

Supplementary Fig. 7 | Steady-state voltammograms of the Au microelectrode in an aqueous solution containing 10 mM $Ru(NH_3)_6Cl_3$ and *x* M Cs_2SO_4 (*x* ranging from 0.05 to 0.15) at 0.02 V s^{-1}. Source data for Supplementary Fig. 7 are provided as a Source Data file.

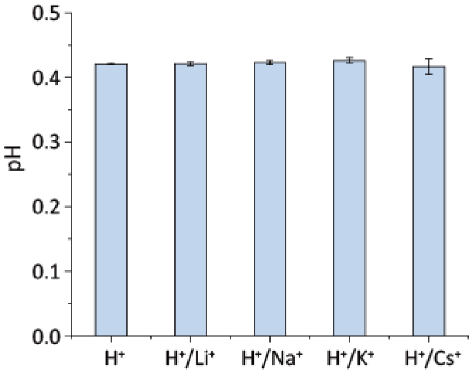

Supplementary Fig. 8 | pH of electrolytes for 0.5 M H_2SO_4 and 0.5 M H_2SO_4 + 0.3 M A^+ (A = Li, Na, K, or Cs). The data is presented as mean ± s.d. (n ≥ 3). Source data for Supplementary Fig. 8 are provided as a Source Data file.

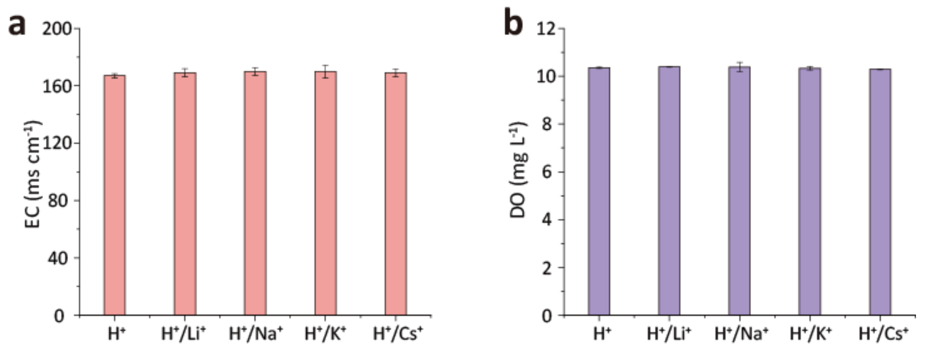

Supplementary Fig. 9 | (a) Electrolyte ionic conductivity (EC) and (b) electrolyte dissolved oxygen (DO) for 0.5 M H_2SO_4 and 0.5 M H_2SO_4 + 0.3 M A^+ (A = Li, Na, K, or Cs). Data in a and b is presented as mean ± s.d. (n ≥ 3). Source data for Supplementary Fig. 9 are provided as a Source Data file.

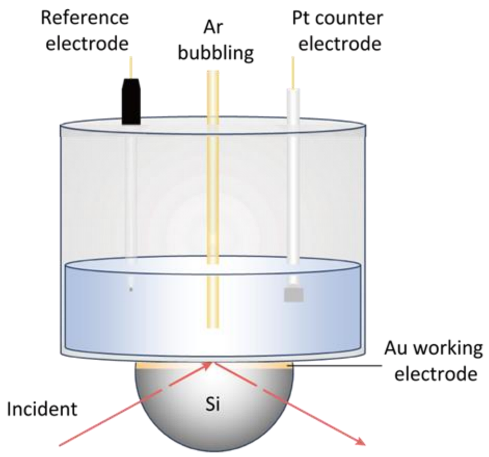

Supplementary Fig. 10 | Schematic showing the set-up for *in situ* ATR-SEIRAS measurement.

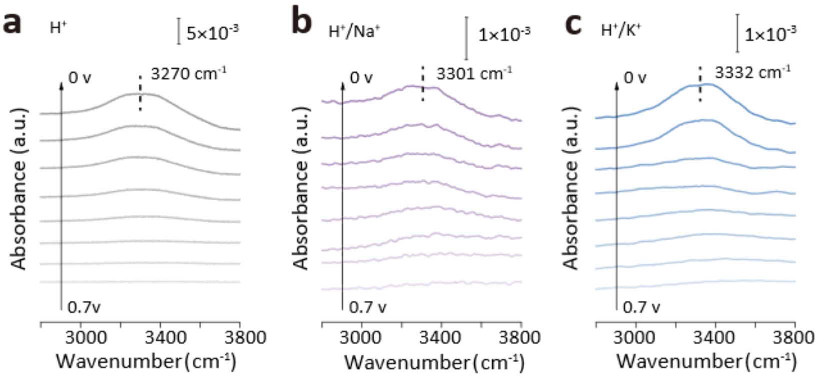

Supplementary Fig. 11 | Potential-dependent OH stretching features of *in situ* ATR-SEIRAS spectra in electrolytes: (a) 0.5 M H_2SO_4 and (b,c) 0.5 M H_2SO_4 + 0.15 M A_2SO_4 (A = Na or K). Source data for Supplementary Fig. 11 are provided as a Source Data file.

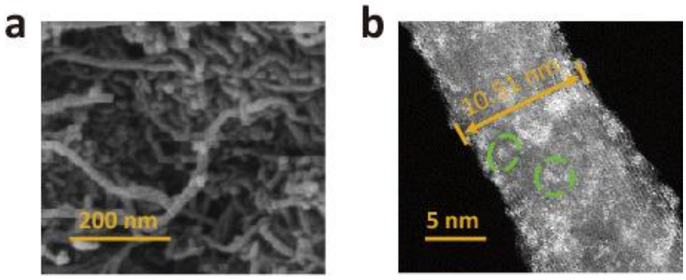

Supplementary Fig. 12 | (a) SEM and (b) AC-HAADF-STEM images of prepared Co-CNT, with green circles highlighting the Co atomic clusters.

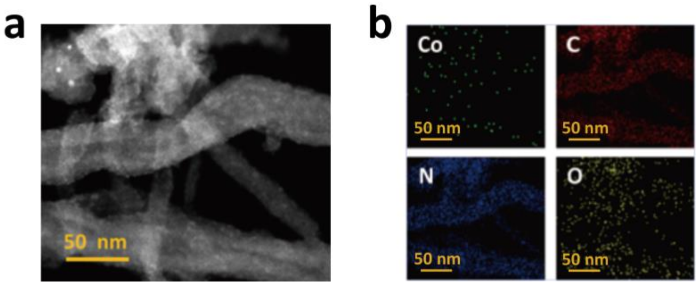

Supplementary Fig. 13 | (a) HAADF-STEM image and (b) corresponding EDS maps of Co-CNT, which verified the homogeneous distribution of C, N, O, and Co elements in the Co-CNT sample.

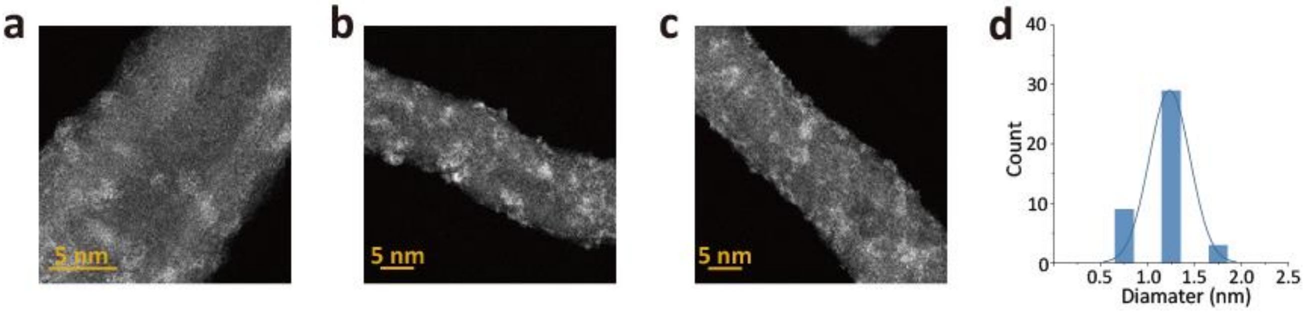

Supplementary Fig. 14 | (a-c) AC-HAADF-STEM images and (d) Co cluster size distribution of the Co-CNT. Source data for Supplementary Fig. 14 are provided as a Source Data file.

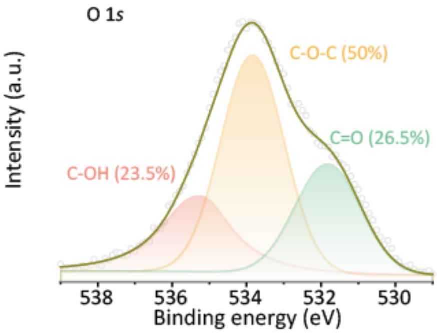

Supplementary Fig. 15 | XPS O 1*s* spectrum of Co-CNT, which could be deconvoluted into three contributions, including C=O (531.8 eV), C-O-C (533.5 eV), and C-OH (535.5 eV). Such XPS measurements confirmed the abundance of bridge oxygen functional groups on Co-CNT, which was consistent with the FT-IR results[1,2]. Source data for Supplementary Fig. 15 are provided as a Source Data file.

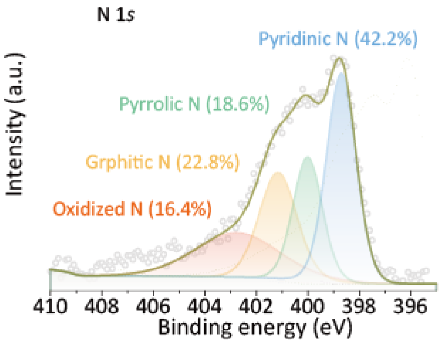

Supplementary Fig. 16 | XPS N 1*s* spectrum of Co-CNT, which could be deconvoluted into four contributions, including oxidized N (402.9 eV), graphitic N (401.3 eV), pyrrolic N (400.1 eV), and pyridinic N (398.5 eV). Such XPS measurements confirmed the abundance of bridge oxygen functional groups at Co-CNT[3]. Source data for Supplementary Fig. 16 are provided as a Source Data file.

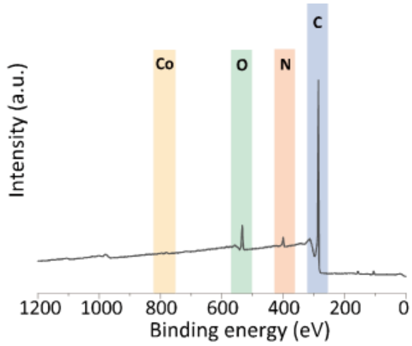

Supplementary Fig. 17 | XPS survey spectrum of Co-CNT. Source data for Supplementary Fig. 17 are provided as a Source Data file.

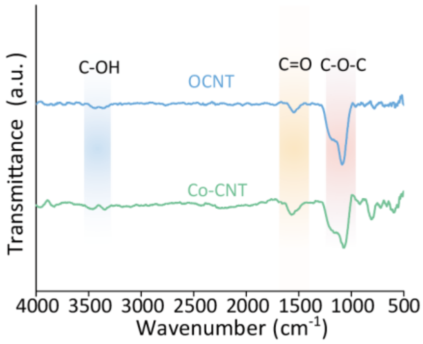

Supplementary Fig. 18 | FTIR spectra of Co-CNT and OCNT, which displayed bands at 1100 cm^{-1} (C-O-C), 1600 cm^{-1} (C=O), and 3450 cm^{-1} (C-OH). The results indicated that the oxygen on Co-CNT mainly existed in the form of C-O-C[4]. Source data for Supplementary Fig. 18 are provided as a Source Data file.

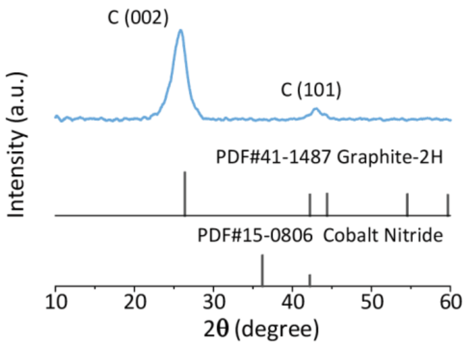

Supplementary Fig. 19 | XRD pattern of Co-CNT, which exhibited two distinct signals at ~26° and 43°. This could be attributed to the (002) and (101) planes of graphite carbon. The comparison with the PDF standard card of cobalt nitride revealed there were no obvious peaks, indicating the absence of cobalt nitride in Co-CNT[3]. Source data for Supplementary Fig. 19 are provided as a Source Data file.

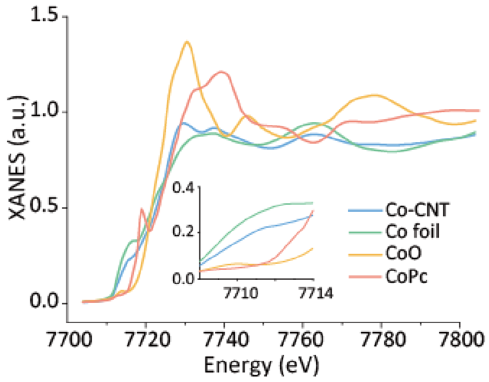

Supplementary Fig. 20 | Co K-edge XANES spectra of Co-CNT and the reference samples (Co foil, CoO and CoPc). The results indicated that the absorption edge energy of Co-CNT was closer to that of Co foil, which proved that the valence of Co was closer to 0[3,5]. Source data for Supplementary Fig. 20 are provided as a Source Data file.

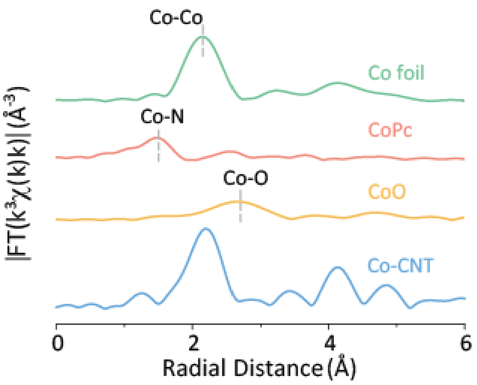

Supplementary Fig. 21 | *k*3-weighted FT-EXAFS spectra of Co-CNT and the reference samples (Co foil, CoO and CoPc). In the FT-EXAFS spectra, Co foil, CoPc, and cobalt suboxide showed distinct FT peaks at 2.14, 1.48, and 2.67 Å, respectively, due to the scattering from Co-Co, Co-N, and Co-O coordination. The FT-EXAFS spectrum of Co-CNT in r-space exhibited a main peak at 2.14 Å, which could be attributed to the metal Co-Co bond in Co nanoclusters. Source data for Supplementary Fig. 21 are provided as a Source Data file.

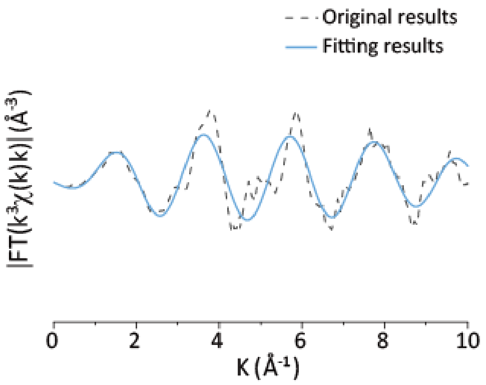

Supplementary Fig. 22 | Co K-edge EXAFS fitting curves of Co-CNT at *k* space. Source data for Supplementary Fig. 22 are provided as a Source Data file.

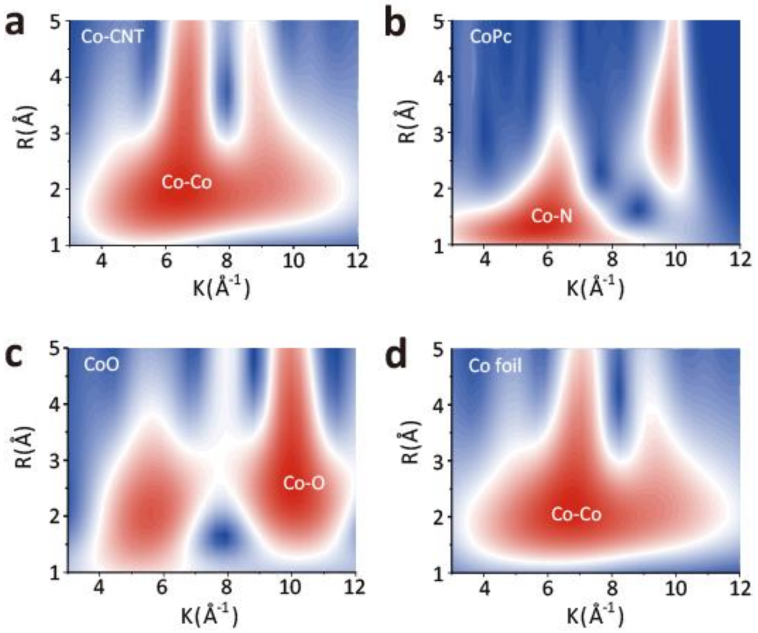

Supplementary Fig. 23 | Co K-edge WT-EXAFS of (a) Co-CNT and (b-d) reference samples. Source data for Supplementary Fig. 23 are provided as a Source Data file.

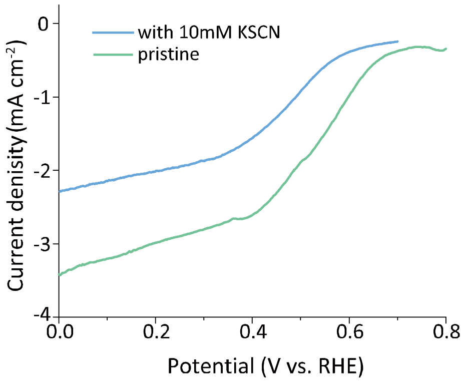

Supplementary Fig. 24 | LSV curves of Co-CNT in O_2-saturated 0.5 M H_2SO_4 + 0.15 M Cs_2SO_4 with and without 10 mM KSCN. The result showed a significant decline of the disk current density, indicating that the Co was indeed the active site for the 2e$^-$ ORR. Source data for Supplementary Fig. 24 are provided as a Source Data file.

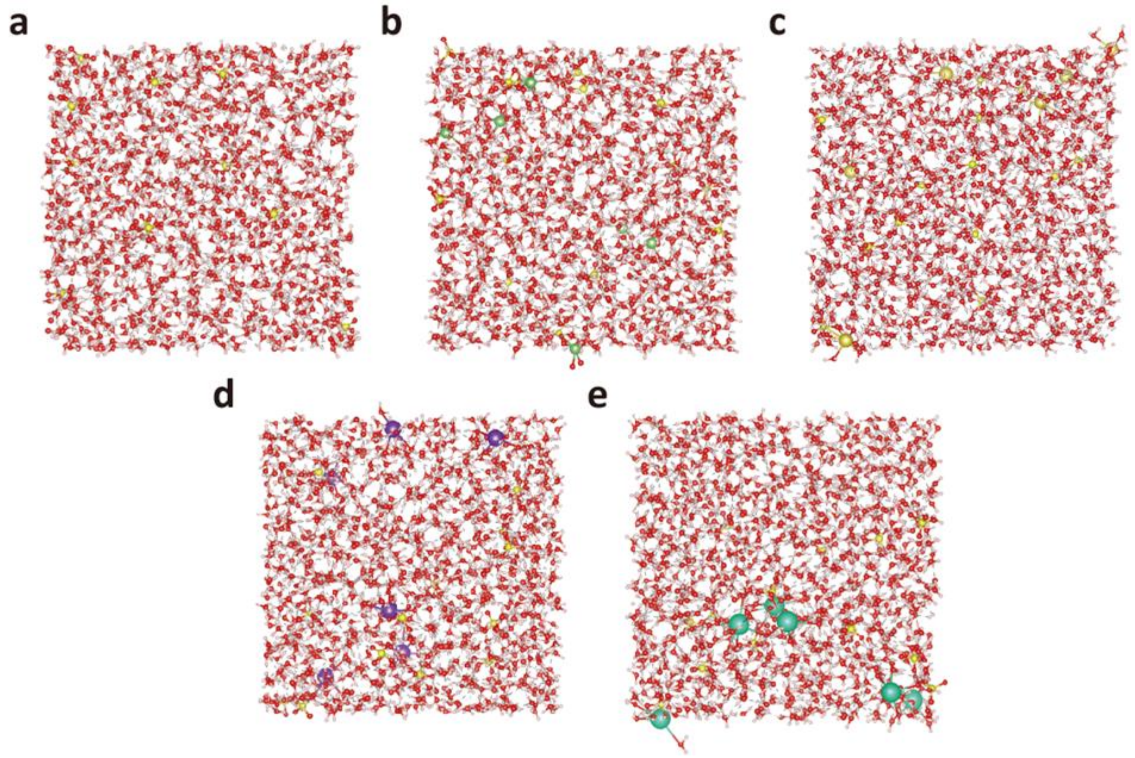

Supplementary Fig. 25 | The MD model employed to quantify hydrogen bonding populations utilized cubic simulation boxes with a side length of 32 Å. The system was populated with hydronium ions, sulfate anions, water molecules, and AMCs (Li^{+}/Na^{+}/K^{+}/Cs^{+}) to simulate the desired electrochemical interface environment. The balls represent H (white), O (red), S (yellow), Li (green), Na (gold), K (purple) and Cs (cyan) atoms. Source data for Supplementary Fig. 25 are provided as Supplementary Data 1.

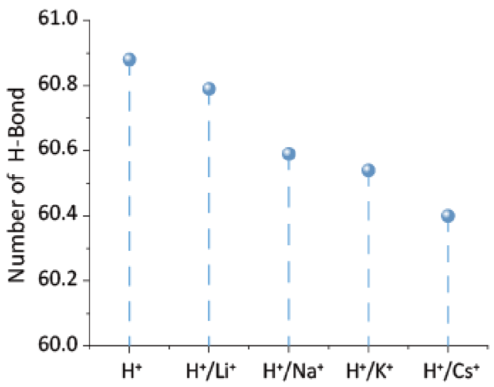

Supplementary Fig. 26 | The number of hydrogen bonds in acid solution with/without AMC. Source data for Supplementary Fig. 26 are provided as a Source Data file.

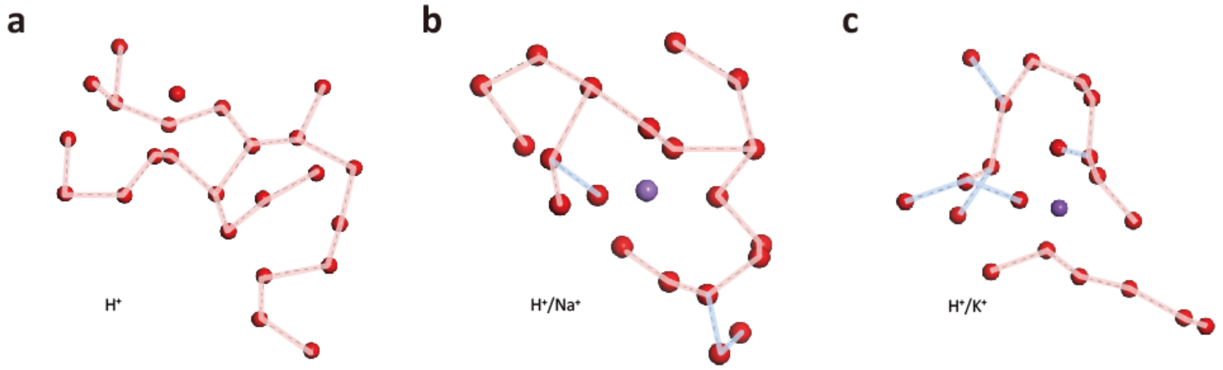

Supplementary Fig. 27 | Hydrogen bonding network connectivity extracted from the results of PIMD for the (a) acid solutions without AMCs, with (b) Na^+ and (c) K^+. For clarity, hydrogen atoms are hidden. Oxygen atoms are shown as red balls. Hydrogen bonds are represented by dashed lines. The balls represent water (red) and Na^+/K^+ (purple) atoms. The pink and blue dashed lines represent the long and short pathways for proton transfer, respectively. Source data for Supplementary Fig. 27 are provided as Supplementary Data 1.

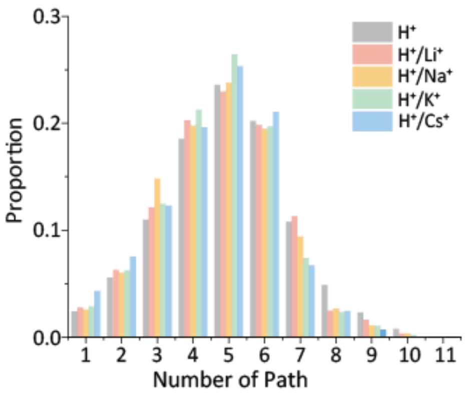

Supplementary Fig. 28 | The path number of hydrogen bonds in acid solution with/without AMC. Source data for Supplementary Fig. 28 are provided as a Source Data file.

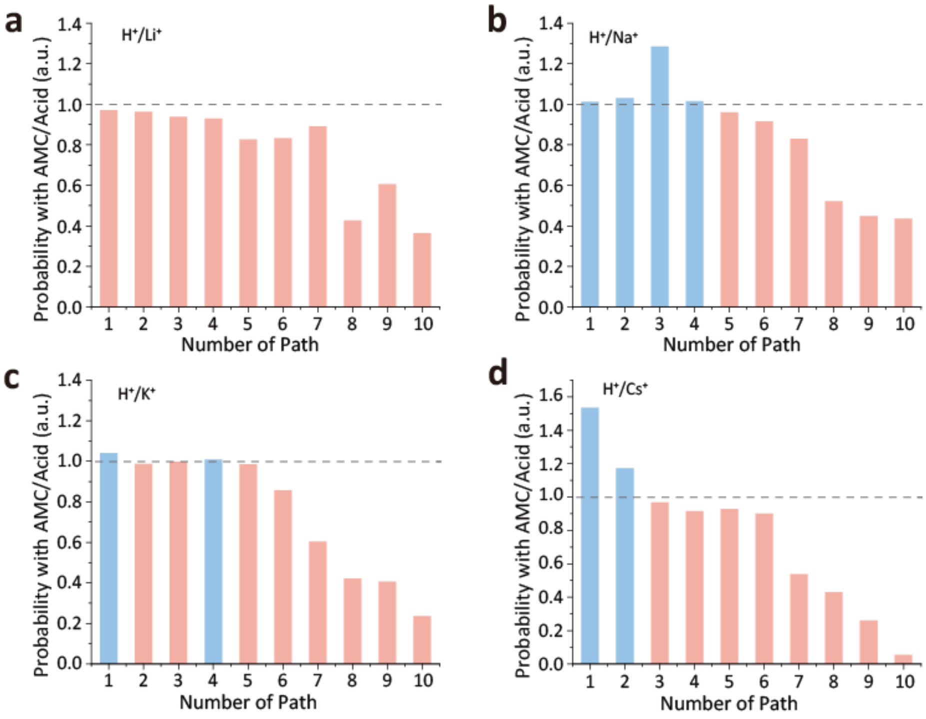

Supplementary Fig. 29 | Relative probabilities of path number of hydrogen bonds in acid solution with AMCs to pure acid solution. Water networks in pure acid solution exhibit higher connectivity than that with AMCs in the PIMD simulation. Source data for Supplementary Fig. 29 are provided as a Source Data file.

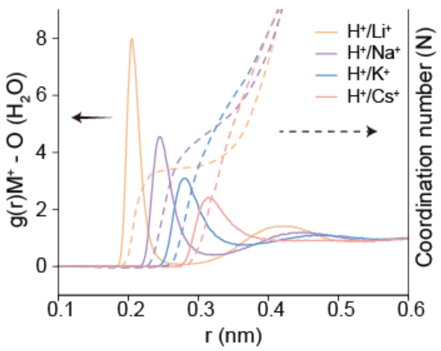

Supplementary Fig. 30 | Radial distribution function (solid lines) and coordination numbers (dashed lines) of solvated metal cation ions and O atoms of H_2O molecules. Source data for Supplementary Fig. 30 are provided as a Source Data file.

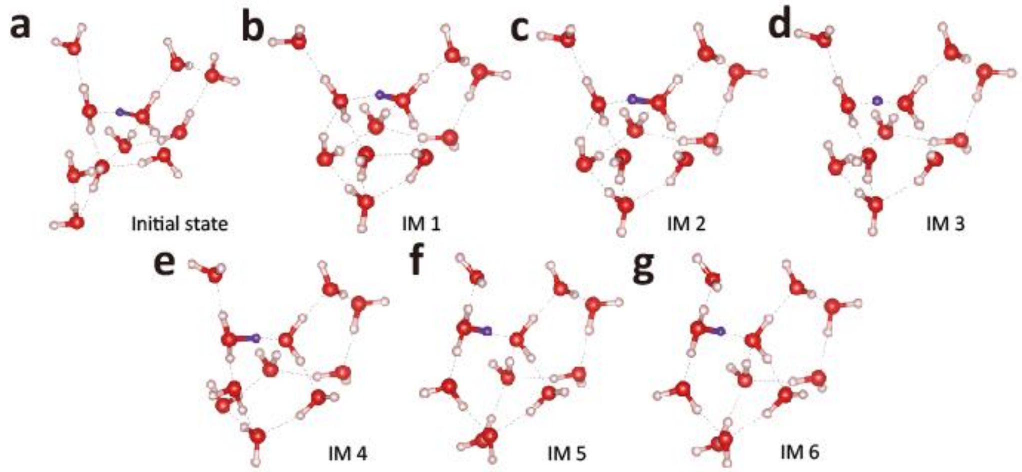

Supplementary Fig. 31 | Snapshots of the initial state and the transition state for proton transfer within the Type I system. The balls represent H (white), O (red) atoms and transferring protons (purple). Source data for Supplementary Fig. 31 are provided as Supplementary Data 1.

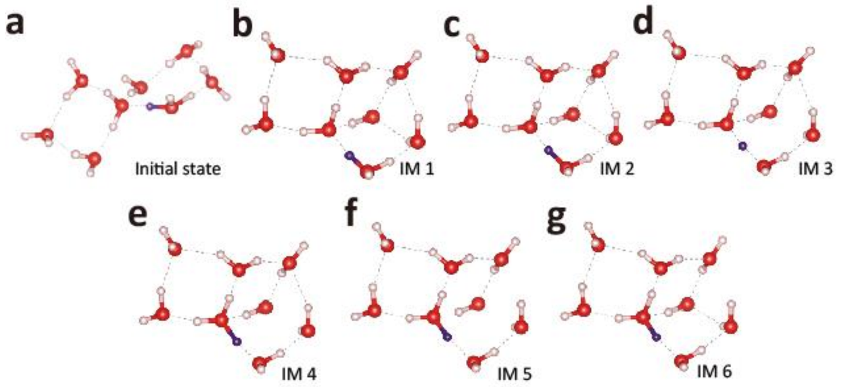

Supplementary Fig. 32 | Snapshots of the initial state and the transition state for proton transfer within the Type II system. The balls represent H (white), O (red) atoms and transferring protons (purple). Source data for Supplementary Fig. 32 are provided as Supplementary Data 1.

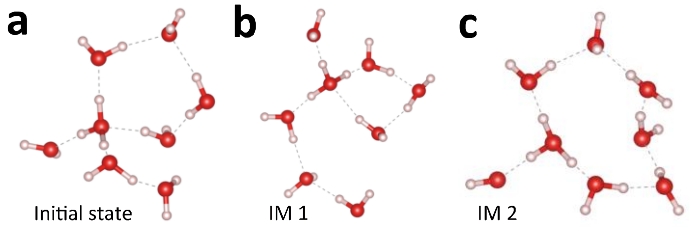

Supplementary Fig. 33 | Snapshots of the initial state and the transition state for proton transfer within the Type III system. The balls represent H (white) and O (red) atoms. Source data for Supplementary Fig. 33 are provided as Supplementary Data 1.

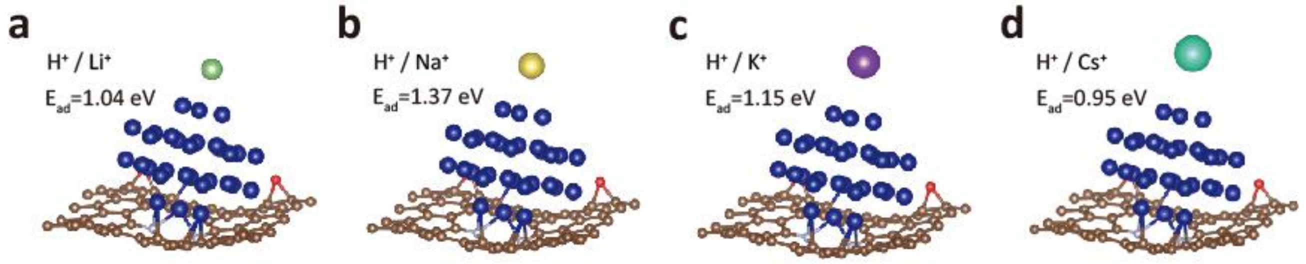

Supplementary Fig. 34 | Optimized structures of anhydrous AMCs on Co-CNT and its adsorption energy, respectively. The balls represent C (brown), N (silver), O (red), Co (blue), Li (green), Na (gold), K (purple) and Cs (cyan) atoms. Source data for Supplementary Fig. 34 are provided as Supplementary Data 1.

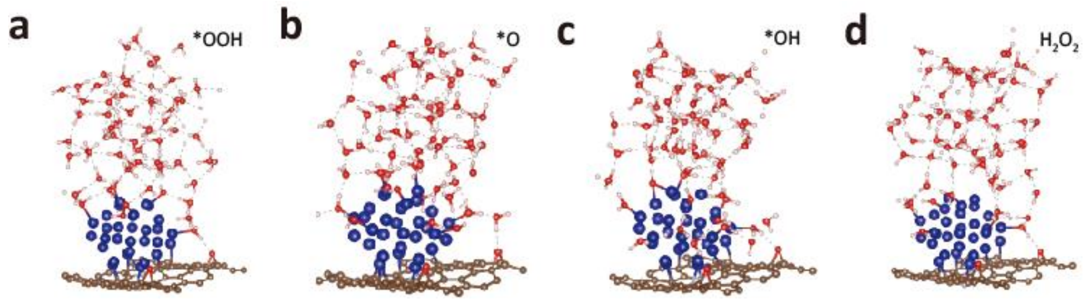

Supplementary Fig. 35 | Calculations were performed to construct free energy diagrams for the hydrogen peroxide electrosynthesis pathway in an acidic electrolyte. Stable structures of key adsorbed intermediates are shown, including *OOH, *O, *OH, and H_2O_2, (asterisks denote adsorption on the catalyst surface). The balls represent C (brown), H (white), N (silver), O (red) and Co (blue) atoms. Source data for Supplementary Fig. 35 are provided as Supplementary Data 1.

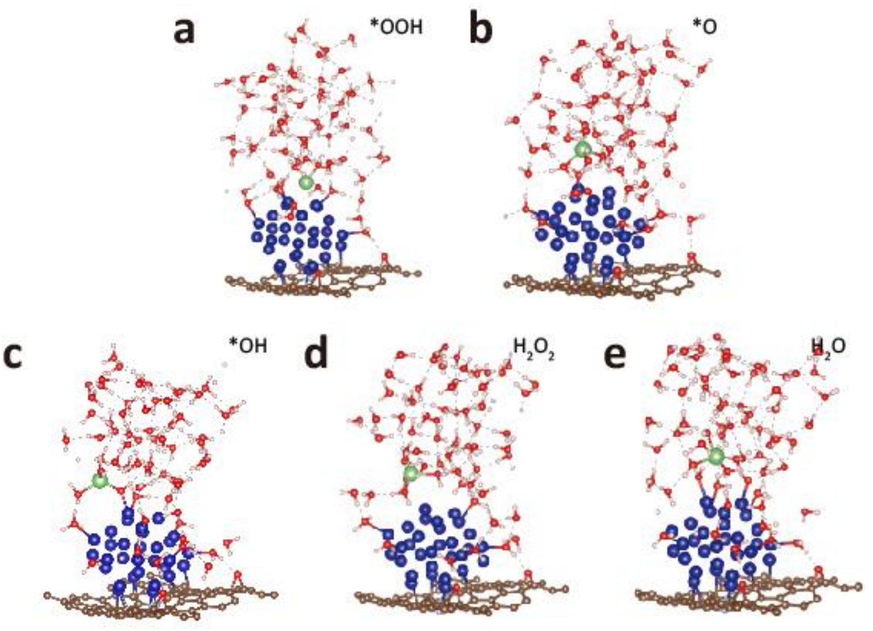

Supplementary Fig. 36 | Calculations were performed to construct free energy diagrams for the hydrogen peroxide electrosynthesis pathway in a Li^+-containing acidic electrolyte. Stable structures of key adsorbed intermediates are shown, including *OOH, *O, *OH, H_2O_2, and H_2O (asterisks denote adsorption on the catalyst surface). The balls represent C (brown), H (white), N (silver), O (red), Co (blue) and Li (green) atoms. Source data for Supplementary Fig. 36 are provided as Supplementary Data 1.

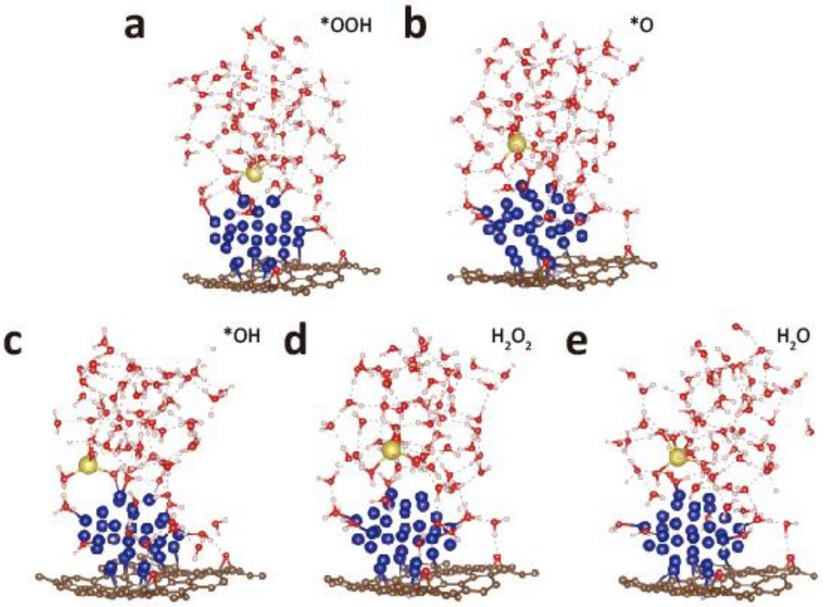

Supplementary Fig. 37 | Calculations were performed to construct free energy diagrams for the hydrogen peroxide electrosynthesis pathway in a Na^+-containing acidic electrolyte. Stable structures of key adsorbed intermediates are shown, including O_2, *OOH, *O, *OH, H_2O_2, and H_2O (asterisks denote adsorption on the catalyst surface). The balls represent C (brown), H (white), N (silver), O (red), Co (blue) and Na (gold) atoms. Source data for Supplementary Fig. 37 are provided as Supplementary Data 1.

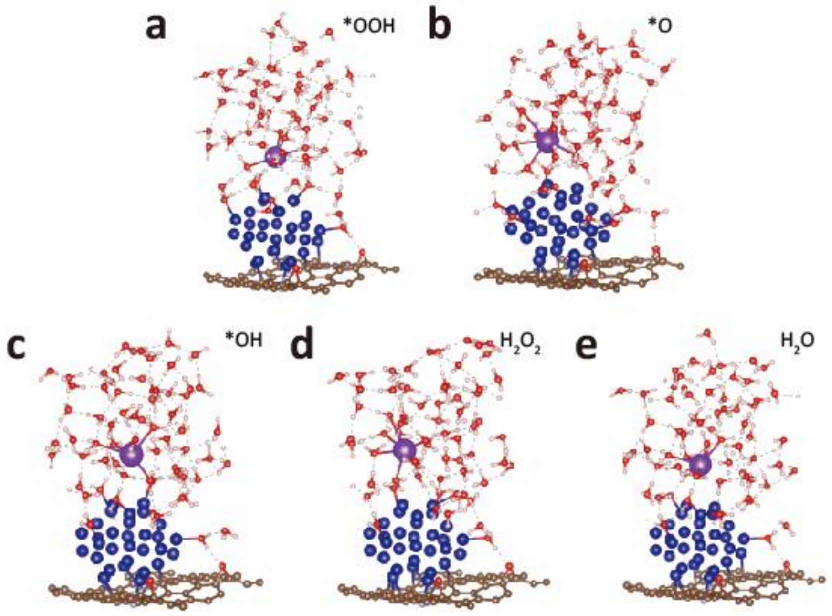

Supplementary Fig. 38 | Calculations were performed to construct free energy diagrams for the hydrogen peroxide electrosynthesis pathway in a K^+-containing acidic electrolyte. Stable structures of key adsorbed intermediates are shown, including *OOH, *O, *OH, H_2O_2, and H_2O (asterisks denote adsorption on the catalyst surface). The balls represent C (brown), H (white), N (silver), O (red), Co (blue) and K (purple) atoms. Source data for Supplementary Fig. 38 are provided as Supplementary Data 1.

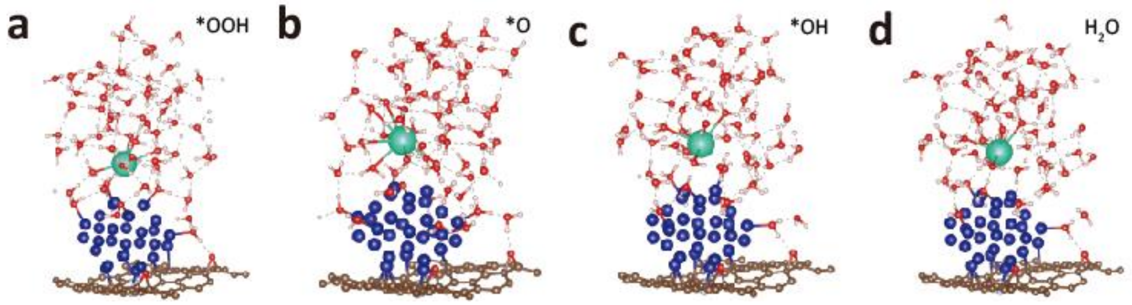

Supplementary Fig. 39 | Calculations were performed to construct free energy diagrams for the hydrogen peroxide electrosynthesis pathway in a Cs^+-containing acidic electrolyte. Stable structures of key adsorbed intermediates are shown, including *OOH, *O, *OH, and H_2O (asterisks denote adsorption on the catalyst surface). The balls represent C (brown), H (white), N (silver), O (red), Co (blue) and Cs (cyan) atoms. Source data for Supplementary Fig. 39 are provided as Supplementary Data 1.

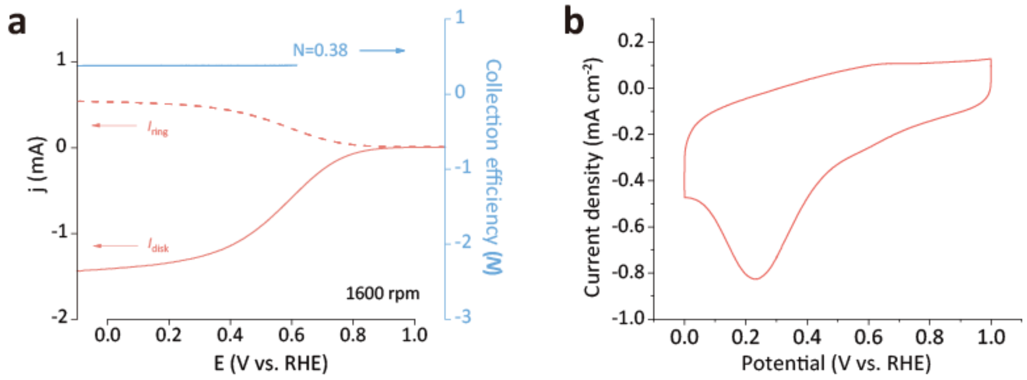

Supplementary Fig. 40 | (a) Calibration of collection efficiency (*N*) of Pt ring electrode in RRDE electrode. The collection efficiency (N) of the RRDE calculated from LSV polarization curves scanned in N_2-saturated 0.1 M KOH electrolyte containing 10 mM potassium ferricyanide ($K_3[Fe(CN)_6]$) using the equation: $N= -I_{ring} / I_{disk}$, where I_{ring} and I_{disk} are the current collected on the Pt ring and glassy carbon disk electrode, respectively. The collection efficiency of RRDE was determined to be 0.38. (b) CV curves of Co-CNT at 10 mV s^{-1} in O_2-saturated 0.5 M H_2SO_4 (area: 0.247 cm^2). Source data for Supplementary Fig. 40 are provided as a Source Data file.

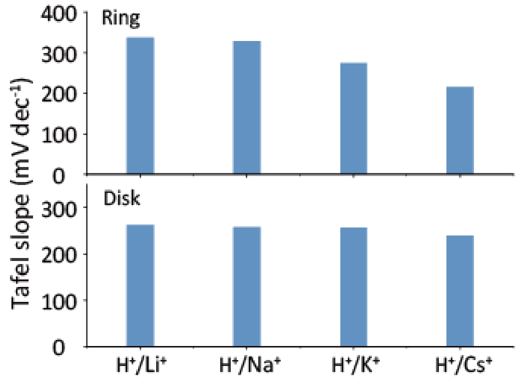

Supplementary Fig. 41 | Tafel slopes of Co-CNT from ring and disk electrodes in electrolytes: 0.5 M H_2SO_4 + 0.15 M A_2SO_4 (A = Li, Na, K, or Cs). Source data for Supplementary Fig. 41 are provided as a Source Data file.

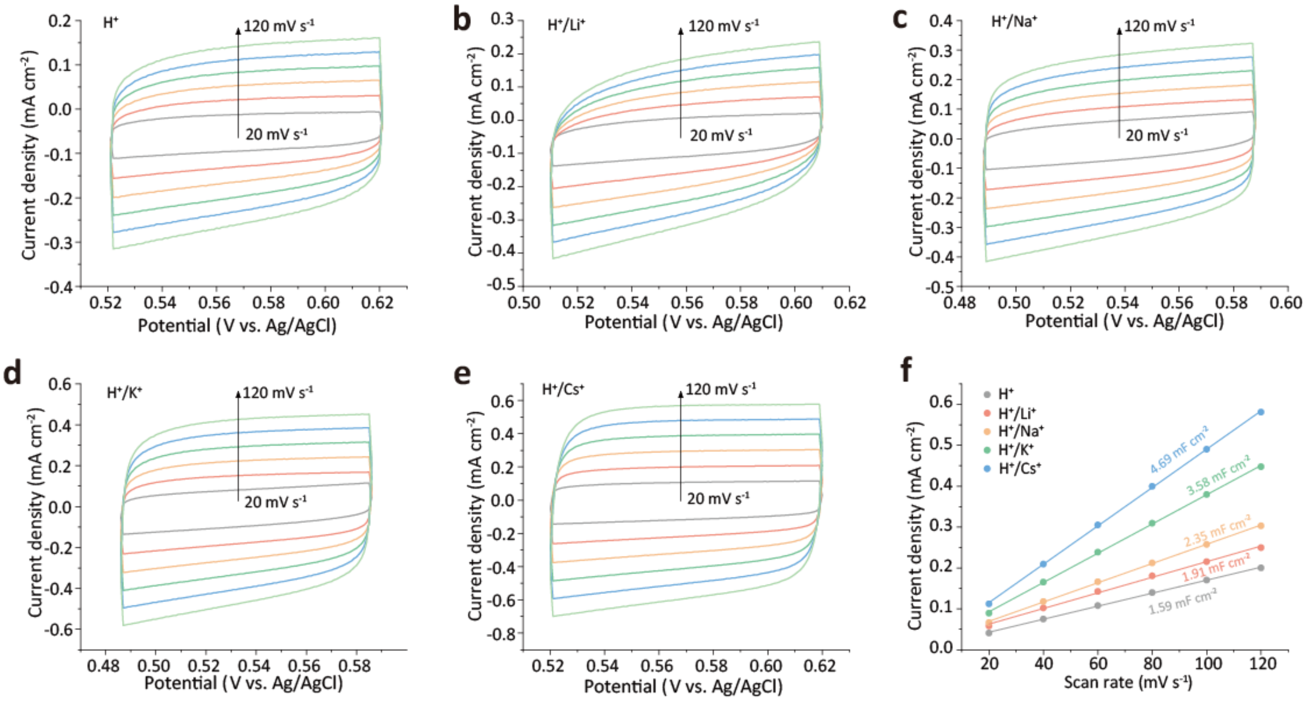

Supplementary Fig. 42 | (a-e) CV curves of Co-CNT at 20-120 mV s^{-1} in N_2-saturated 0.5 M H_2SO_4 and 0.5 M H_2SO_4 + 0.15 M A_2SO_4 (A = Li, Na, K, Cs). (f) Calculated C_{dl} of the Co-CNT based on CV curves at different sweep rates. Source data for Supplementary Fig. 42 are provided as a Source Data file.

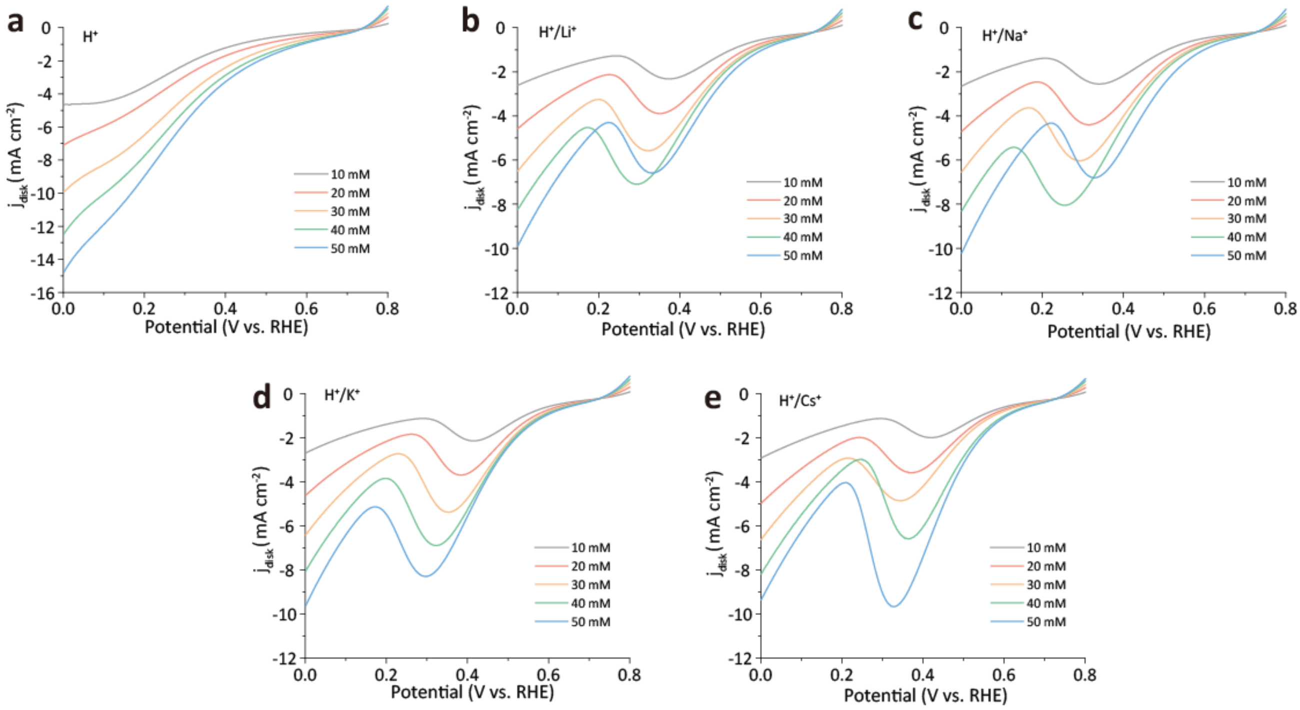

Supplementary Fig. 43 | H_2O_2RR polarization curves of Co-CNT in N_2-saturated (a) 0.5 M H_2SO_4 and (b-e) 0.5 M H_2SO_4 + 0.15 M A_2SO_4 (A = Li, Na, K, Cs) containing different concentrations of H_2O_2. Source data for Supplementary Fig. 43 are provided as a Source Data file.

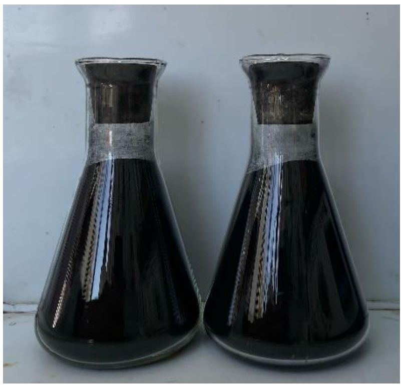

Supplementary Fig. 44 | Digital photograph showing the batch production of the Co-CNT catalyst.

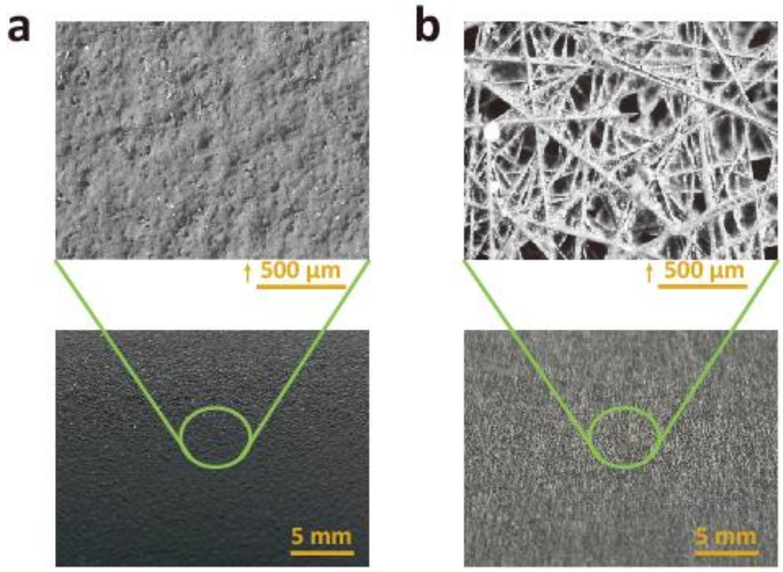

Supplementary Fig. 45 | SEM images of the gas diffusion electrode (GDE) and its surface morphology. (a) Catalyst layer and (b) Gas diffusion layer.

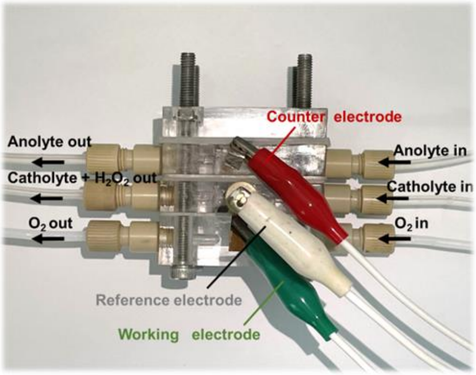

Supplementary Fig. 46 | Photo of flow-cell reactor for electrochemical H_2O_2 production.

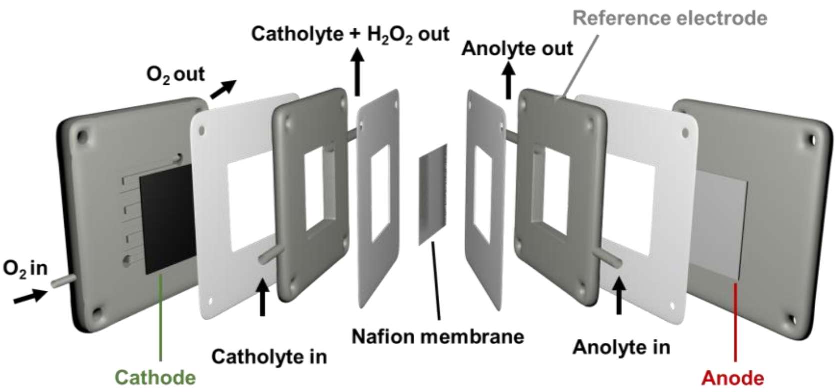

Supplementary Fig. 47 | Schematic diagram of the flow cell for H_2O_2 production.

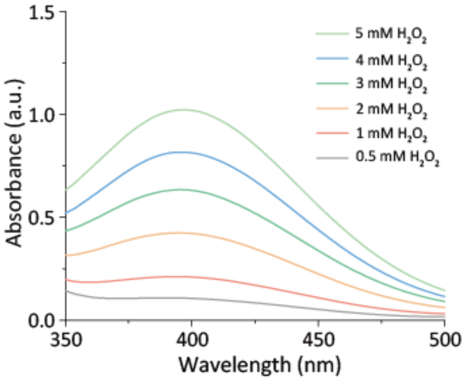

Supplementary Fig. 48 | UV–vis absorption spectra of different concentrations of standard H_2O_2 solutions titrated with potassium titan oxalate. Source data for Supplementary Fig. 48 are provided as a Source Data file.

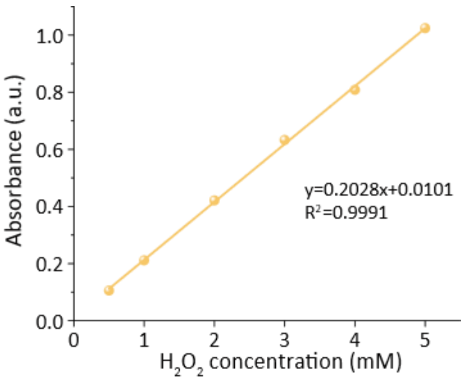

Supplementary Fig. 49 | The linear absorbance–concentration calibration curve at 400 nm. Source data for Supplementary Fig. 49 are provided as a Source Data file.

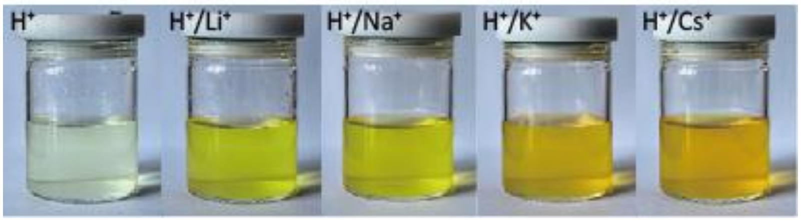

Supplementary Fig. 50 | Electrolytes with potassium titanium (IV) oxalate solution after 1 h operation in all five electrochemical systems.

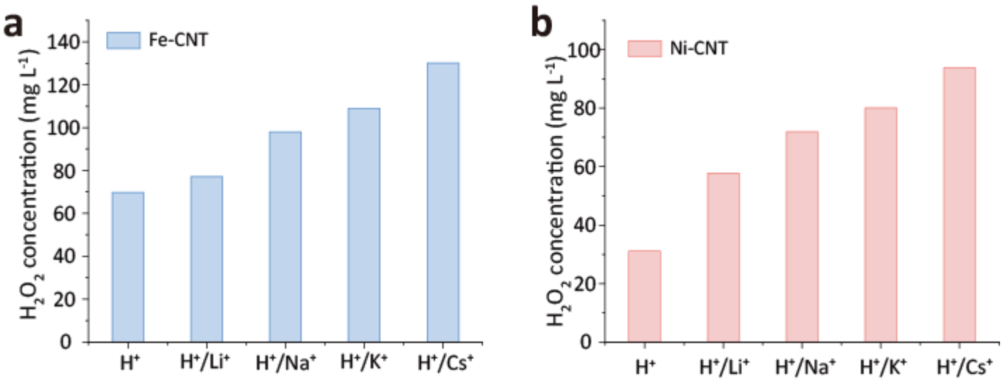

Supplementary Fig. 51 | The H_2O_2 yield of (a) Fe-CNT and (b) Ni-CNT in O_2-saturated 0.5 M H_2SO_4 and 0.5 M H_2SO_4 + 0.15 M A_2SO_4 (A = Li, Na, K, Cs) in a flow-cell reactor. Source data for Supplementary Fig. 51 are provided as a Source Data file.

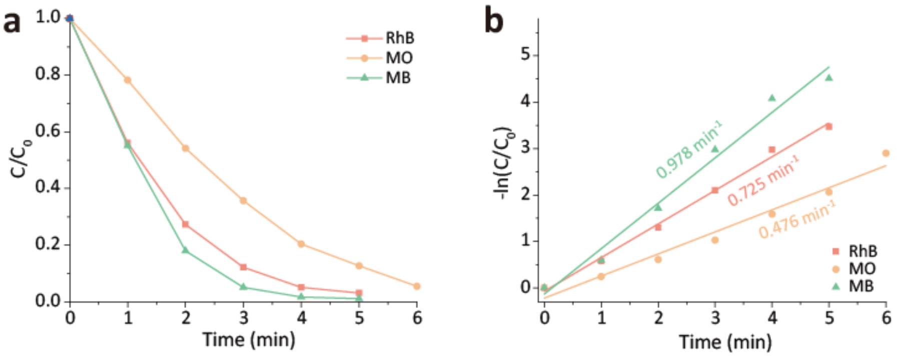

Supplementary Fig. 52 | (a) Fenton degradation of 10 mg L^{-1} dyes and (b) k-value of pollutant degradation in flow cell. Source data for Supplementary Fig. 52 are provided as a Source Data file.

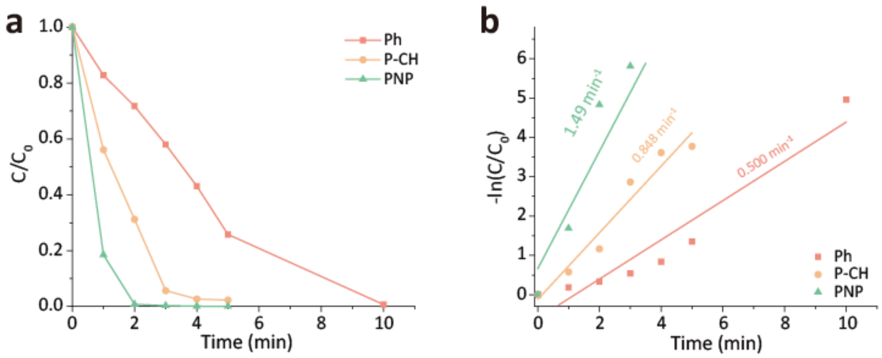

Supplementary Fig. 53 | (a) Fenton degradation of 1 mg L^{-1} aromatic pollutants and (b) k-value of pollutant degradation in flow cell. Source data for Supplementary Fig. 53 are provided as a Source Data file.

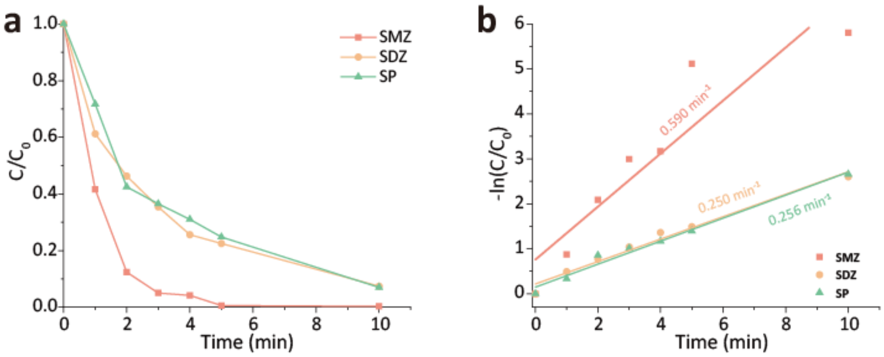

Supplementary Fig. 54 | (a) Fenton degradation of 1 mg L^{-1} sulfonamide pollutants and (b) k-value of pollutant degradation in flow cell. Source data for Supplementary Fig. 54 are provided as a Source Data file.

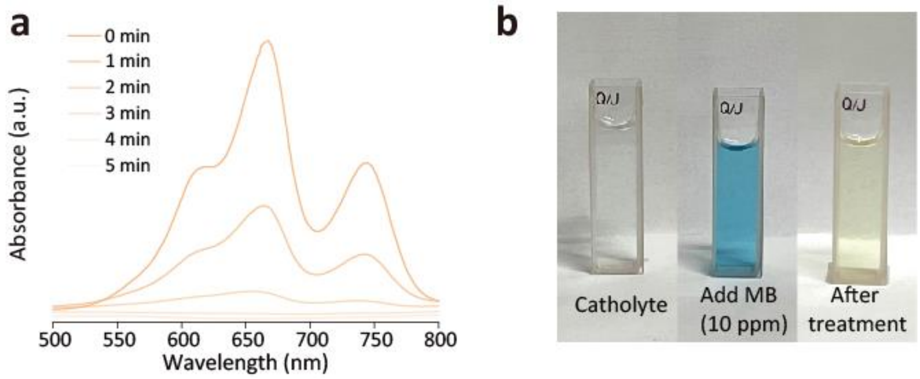

Supplementary Fig. 55 | (a) UV–vis absorption spectra of methylene blue (MB) removal status and (b) digital photo of the catholyte (left panel), catholyte with 10 mg L^{-1} MB (middle panel), and catholyte with 10 mg L^{-1} MB and 10 mM Fe^{2+} (right panel). Source data for Supplementary Fig. 55 are provided as a Source Data file.

Supplementary Table

Supplementary Table 1 | Co K-edge EXAFS fitting parameters for Co-CNT.

Name	Path	C.N.	$10^3 * \sigma^2$ ($Å^2$)	ΔE_0 (eV)	R (Å)	R-factor
Co foil	Co-Co	12	2.49	0.00521	6.8	0.03
Co-CNT	Co-Co	7.442	1.52	14.795	2.274	0.025
	Co-N	3.167	5.79		1.929	0.020

Supplementary Table 2 | The derived properties of simulated solution.

System	Number (H_2O)	Length (Å)	ρ (g/cm^3)	Number (H-bond)	N/V (nm^{-3})
H^+	1085	32.15	1.04	2024	60.88
H^+/Li^+	1084	32.18	1.06	2025	60.79
H^+/Na^+	1079	32.15	1.06	2014	60.59
H^+/K^+	1076	32.15	1.06	2012	60.54
H^+/Cs^+	1071	32.13	1.09	2004	60.40

Supplementary Table 3 | Comparison of H_2O_2 production between this work and reported electrocatalysts via 2e⁻ ORR.

Catalyst	Electrolyte	Current	H_2O_2 production	References
Co-OCNT	0.5 M H_2SO_4 /0.15 M Cs_2SO_4	500	9.26	This work
	0.5 M H_2SO_4 /0.15 M K_2SO_4	500	7.67	This work
	0.5 M H_2SO_4 /0.15 M	500	5.76	This work
CoIn-N-C	0.1 M $HClO_4$	100	9.68	6
Co-N_5/C	0.5 M H_2SO_4	15	6.78	7
Co SA-AC/CNT	0.5 M H_2SO_4	300	3.88	4
BBL-PcNi	0.1 M $HClO_4$	100	5.80	8
Se_2-Pt NPs	0.1 M $HClO_4$	250	4.16	9
CoN_4/VG	0.1 M $HClO_4$	21	4.00	10
PtP_2	0.1 M $HClO_4$	150	2.60	11
$Pd^{\delta+}$-OCNT	0.1 M $HClO_4$	10	1.70	12
$CoSe_2$	0.5 M H_2SO_4	63	0.90	13
h-Pt1-CuSx	0.5 M $HClO_4$	10	0.54	14

Supplementary Reference

1 Chen, Y. *et al.* Oxygen Functional Groups Regulate Cobalt-Porphyrin Molecular Electrocatalyst for Acidic H_2O_2 Electrosynthesis at Industrial-Level Current. *Angew. Chem. Int. Ed.* **63**, e202407163 (2024).

2 Gu, Y. *et al.* Industrial electrosynthesis of hydrogen peroxide over p-block metal single sites. *Nat. Synth.* **4**, 614-621 (2025).

3 Yu, K. *et al.* Engineering Asymmetric Electronic Structure of Co-N-C Single-Atomic Sites Toward Excellent Electrochemical H_2O_2 Production and Biomass Upgrading. *Angew. Chem. Int. Ed.* **64**, e202502383 (2025).

4 Zhang, H. *et al.* Harmonizing Cobalt Atom-Cluster Moieties with Selective Oxygen Functional Groups for Augmented Electrocatalytic H_2O_2 Production. *Adv. Funct. Mater.* **35**, 2417090 (2025).

5 Qi, D. *et al.* Cyclodextrin-supported $Co(OH)_2$ Clusters as Electrocatalysts for Efficient and Selective H_2O_2 Synthesis. *Angew. Chem. Int. Ed.* **62**, e202307355 (2023).

6 Du, J. *et al.* CoIn dual-atom catalyst for hydrogen peroxide production via oxygen reduction reaction in acid. *Nat. Commun.* **14**, 4766 (2023).

7 Yan, L. *et al.* Optimizing the binding of the *OOH intermediate via axially coordinated $Co-N_5$ motif for efficient electrocatalytic H_2O_2 production. *Appl. Catal. B-Environ.* **338**, 123078 (2023).

8 Tang, B., Pauls, M., Bannwarth, C. & Hecht, S. Photoswitchable Quadruple Hydrogen-Bonding Motif. *J. Am. Chem. Soc.* **146**, 45-50 (2024).

9 Yu, Z. *et al.* Selective and durable H_2O_2 electrosynthesis catalyst in acid by selenization induced straining and phasing. *Nat. Commun.* **15**, 9346 (2024).

10 Lin, Z. *et al.* Atomic Co decorated free-standing graphene electrode assembly for efficient hydrogen peroxide production in acid. *Energy Environ. Sci.* **15**, 1172-1182 (2022).

11 Li, H. *et al.* Scalable neutral H_2O_2 electrosynthesis by platinum diphosphide nanocrystals by regulating oxygen reduction reaction pathways. *Nat. Commun.* **11**, 3928 (2020).

12 Chang, Q. *et al.* Promoting H_2O_2 production via 2-electron oxygen reduction by coordinating partially oxidized Pd with defect carbon. *Nat. Commun.* **11**, 2178 (2020).

13 Zhang, X.-L. *et al.* Strongly Coupled Cobalt Diselenide Monolayers for Selective Electrocatalytic Oxygen Reduction to H_2O_2 under Acidic Conditions. *Angew. Chem. Int. Ed.* **60**, 26922-26931 (2021).

14 Shen, R. *et al.* High-Concentration Single Atomic Pt Sites on Hollow CuS_x for Selective O_2 Reduction to H_2O_2 in Acid Solution. *Chem* **5**, 2099-2110 (2019).
