# Supplementary material for: Differentiating interfacial water structures via alkali metal cation promotor for H2O2 electrosynthesis in acid
Source: Nat Commun. 2026 Apr 8;17:4973. doi: 10.1038/s41467-026-71584-9 (PMC13237355; doi:10.1038/s41467-026-71584-9)
Supplement: Supplementary file 2 — Description of Additional Supplementary Files [file 41467_2026_71584_MOESM2_ESM.pdf]

Description of Additional Supplementary Files

File Name: Supplementary Data 1

Description: This file includes all the atomic coordinates of the optimized computational models, i. e.

- Cs-E_{ad}.vasp
- Cs-H_2O.vasp
- Cs-H_2O_2.vasp
- Cs-MD-final.vasp
- Cs-MD-initial.vasp
- Cs-O.vasp
- Cs-OH.vasp
- Cs-OOH.vasp
- H-H_2O.vasp
- H-H_2O_2.vasp
- H-MD-final.vasp
- H-MD-initial.vasp
- H-O.vasp
- H-OH.vasp
- H-OOH.vasp
- K-E_{ad}.vasp
- K-H_2O.vasp
- K-H_2O_2.vasp
- K-MD-final.vasp
- K-MD-initial.vasp
- K-O.vasp
- K-OH.vasp
- K-OOH.vasp
- Li-E_{ad}.vasp
- Li-H_2O.vasp
- Li-H_2O_2.vasp
- Li-MD-final.vasp
- Li-MD-initial.vasp
- Li-O.vasp
- Li-OH.vasp
- Li-OOH.vasp
- Na-E_{ad}.vasp
- Na-H_2O.vasp
- Na-H_2O_2.vasp
- Na-MD-final.vasp
- Na-MD-initial.vasp
- Na-OOH.vasp
- Na-O.vasp
- Na-OH.vasp
